# Editorial: Metabolic engineering for the production of bioactive compounds

**DOI:** 10.3389/fmicb.2026.1822448

**Published:** 2026-03-24

**Authors:** Fabio Marcio Squina, Francisco José Fernández Perrino, Jayesh Jagannath Ahire, Yun-Peng Chao

**Affiliations:** 1 Laboratório de Ciências Moleculares, Universidade de Sorocaba, Sorocaba, Brazil; 2 División de Ciencias Biológicas y de la Salud, Departamento de Biotecnología, Universidad Autónoma Metropolitana, Iztapalapa, Mexico; 3 Dr. Reddy's Laboratories, Hyderabad, India; 4 Department of Chemical Engineering, Feng Chia University, Taichung, Taiwan

**Keywords:** bioactive compounds, biofuel, computational modeling, industrial processes, metabolic engineering

Metabolic engineering and synthetic biology are now central components of modern biotechnology, enabling the rational redesign of microbial metabolism to produce chemicals and bioactive compounds that were traditionally obtained from non-renewable or scarce natural resources (Keasling, 2010). From antibiotics and antifungals to biofuels, specialty chemicals, and cosmeceuticals, engineered microbial cell factories now support diverse industrial sectors (Decker et al., 2023; Shi et al., 2023). This Research Topic highlights advances in microbial systems for supplying the next generation of bioactive agents by decoding natural metabolic pathways and applying advanced biotechnological tools to improve conversion efficiency, boost yields, and achieve industrial scalability.

The review by Yogiswara and Verstrepen summarizes advances in the microbial production of 3-methyl-1-butanol (3MB), a solvent with growing industrial markets and emerging applications as an advanced biofuel and *platform chemical*. Their work provides a comprehensive analysis of branched-chain alcohol biosynthesis in engineered strains, including host strain selection, feedstock diversification, and fermentation design. By comparing the three main *pathways*, based on the valine–leucine–Ehrlich pathway, the mevalonate pathway, and the isovaleryl-CoA pathway, the authors highlight key challenges in developing competitive bioproduction platforms. These challenges include redirecting metabolic flux toward leucine-derived intermediates and reducing byproducts such as isobutanol and ethanol. The review outlines core strategies to address these issues, including engineering feedback-resistant variants of α-isopropylmalate synthase, rebalancing the NAD(P)H cofactors, and optimizing gene expression levels. Furthermore, the review covers process innovations, alongside high-throughput technologies, that have accelerated the development of scalable and economically viable microbial platforms for 3MB production.

Meng et al. identify redox homeostasis as a crucial bottleneck in polyketide biosynthesis, demonstrating that targeted enhancement of NAD(P) metabolism substantially increases natamycin production in *Streptomyces gilvosporeus*. The authors increased natamycin yield by varying the organic nitrogen sources, thereby stimulating the activity of the NAD(P) metabolic pathway. Integrated transcriptomic and metabolomic analyses confirmed the upregulation of genes involved in NAD and NADP biosynthesis, resulting in elevated intracellular redox cofactor levels and increased natamycin production. The authors reinforce the idea that NAD(P) metabolism is a strategic target for rational approaches to polyketide biosynthesis.

The review by Martin et al. investigates the role of the global regulator LaeA in transcription and its coordination of development, metabolite secretion, and environmental adaptation. As a key part of the velvet complex, LaeA influences the production of fungal cellulolytic/ligninolytic enzymes, in addition to citric acid secretion in Ascomycetes. It also regulates numerous secondary metabolites in Ascomycetes, such as *Alternaria* host-specific toxins, T-toxin in *Cochliobolus*, penicillin and PR-toxin in *Penicillium*, and aflatoxins and ochratoxin A in *Aspergillus*. Additionally, LaeA regulates the production of siderophores and bioactive triterpenes in Basidiomycetes, including ganoderic acid in *Ganoderma*. The review also discusses how MAP kinase signaling converges on the velvet complex (LaeA, VeA, VelB) to coordinate activation or repression of specific secondary metabolite gene clusters. Finally, the authors propose that LaeA and the velvet complex engage in epigenetic-like mechanisms, suggesting their potential to coordinate the activation or silencing of these *loci* in response to developmental and environmental cues.

The review by Gluth et al. synthesizes over 40 proteomic studies of oleaginous yeasts and filamentous fungi, offering critical insights into lipid accumulation mechanisms that can be applied to metabolic engineering strategies. The authors clarify the mechanisms linking nitrogen limitation to AMP depletion and citrate overflow, which divert organisms' strategies for generating acetyl-CoA and NADPH. These strategies enhance the pentose phosphate pathway, malic enzyme activity, and alternative redox cycles. Interestingly, lipid droplet proteomic and post-translational analyses highlight multiple pathways involved in lipogenesis in fungi.

The original research by Gaitanis et al. highlights the ongoing importance of exploring microbial diversity to discover new chemical scaffolds. Their research reports screening 980 Greek actinobacterial strains for skin anti-aging activity, identifying metabolites with elastase and tyrosinase inhibitory properties. Through fractionation and characterization, several bioactive molecules were isolated and shown to influence oxidative stress responses and lysosomal activity.

Jain et al. investigated cinnabar (HgS)-induced hormesis to elucidate the stress-mediated metabolic reprogramming of dendrobine biosynthesis through multi-omics profiling. Their studies on *Trichoderma longibrachiatum* MD33 reveal that subtoxic levels of cinnabar enhance dendrobine production via the coordinated upregulation of terpenoid pathways, whereas slightly higher levels of HgS trigger oxidative damage and metabolic repression. This work highlights that stress-induced secondary metabolism is highly non-linear, with a narrow window between activation and collapse, reflecting fundamental constraints on how far secondary metabolism can be pushed without compromising cell redox buffering, antioxidant capacity, and stress-response networks.

Li et al. describe a new exo-lytic glycosaminoglycan lyase from a marine *Microbacterium* that effectively produces unsaturated disaccharides from various sulfated and non-sulfated substrates. Through thorough biochemical studies and docking analyses, they identified potential enzyme applications for producing glycosaminoglycan disaccharides and specific residues that may explain the enzyme's broad substrate range and unique mechanism of action. Their research could support future efforts to discover enzyme resources and rational enzyme design, in addition to improving enzymes relevant to the production of glycosaminoglycan disaccharides, which are important for glycobiology and therapy development.

Finally, Du et al. introduce a novel rare-codon replacement strategy, combined with mutagenesis and metabolic engineering, to efficiently identify and develop high-yield L-valine-producing strains. The authors engineered an artificial selection marker, LESG, by replacing common L-valine codons with the rare GTC codon in target genes. Since translation of rare codons depends on intracellular amino acid availability, fluorescence intensity directly correlates with L-valine production levels. This integrated approach led to the isolation of the *Escherichia coli* DK2 strain, which achieved 84.1 g/L of L-valine in 24 h of bioreactor fermentation.

Collectively, the contributions in this Research Topic demonstrate how metabolic engineering has evolved from a focus on individual pathways to becoming an integrated, systems-level discipline. The manuscripts showcase a wide range of advanced biotechnological tools, such as multi-omics platforms, rare codon-based screening systems, high-throughput selection methods, computational modeling, and genome editing technologies. These approaches have greatly accelerated strain development and improved the translation of laboratory innovations into sustainable, environmentally friendly industrial processes.
